# In Vivo Evaluation of the Acute Systemic Toxicity of (1S,2E,4R,6R,7E,11E)-Cembratriene-4,6-diol (4R) in Sprague Dawley Rats

**DOI:** 10.3390/nutraceuticals2020005

**Published:** 2022-04-09

**Authors:** Nadezhda Sabeva, Oné R. Pagán, Yancy Ferrer-Acosta, Vesna A. Eterović, Peter A. Ferchmin

**Affiliations:** 1 Department of Neuroscience, Medical of School, Universidad Central del Caribe, Bayamón, PR 00956, USA; 2 Department of Biology, West Chester University, 750 S Church St., West Chester, PA 19383, USA; 3 Neuroprotection for Life, Carmel, IN 46032, USA

**Keywords:** cembranoids, subacute toxicity, *Nicotiana tabacum*

## Abstract

The tobacco cembranoid (1S,2E,4R,6R,7E,11E)-2,7,11-cembratriene-4,6-diol (4R) interacts with nicotinic acetylcholine receptors, which results in neuroprotection against organophosphate toxicity, brain ischemia, and Parkinson’s disease. The present study is a continuation of our previous research in which we applied a single dose of 4R 1 h before or 24 h after exposure to diisopropylfluorophosphate (DFP) (analog of the nerve agent sarin). The 4R dose robustly decreased neuroinflammation and neuronal death at both timepoints. Here, we investigated the toxicity of a single dose of 4R in male and female Sprague Dawley (SD) rats after a subcutaneous (s.c.) injection of 6, 24, or 98 mg/kg. Body weight was not affected by 4R during the 7-day observation period. No histopathologic changes in the organs were attributed to 4R. Minor hematological and blood composition variations were detected on Day 3 in the mid- and the high-dose males, but these were resolved by Day 8. At the area of the s.c. injection site, alopecia and dry skin were detected in both the 4R-treated males and females and in the female controls.

## 1. Introduction

Cembranoids are diterpenoids derived from the cembrane diterpene with various degrees of oxygenation and hydroxylation. The most abundant variety of cembranoids has been found in Caribbean and Pacific gorgonians [1–3], but these molecules are also present in terrestrial plants of the *Nicotiana* and *Pinus genera* [4]. *Nicotiana tabacum* cembranoids have become appealing targets in natural product chemistry research [5]. More than eighty tobacco cembranoids have been isolated, but studies concerning their bioactivities have mainly concentrated on two candidates, (1S,2E,4S,7E,11E)-cembra-2,7,11-triene-4,6-diol (4S) and (1S,2E,4R,7E,11E)-cembra-2,7,11-triene-4,6-diol (4R). The 4S and 4R candidates are known to display neuroprotective (4R) [5], antibacterial [6], and anti-tumor properties against breast (4S) and prostate cancer cells (4R) [7–10].

Widely used in industry, agriculture, warfare, and terroristic attacks as nerve agents, acetylcholinesterase (AChE) inhibitors (AChE-I) present a threat to human life. Acute AChE-I poisoning generally causes neurological damage due to the synaptic accumulation of acetylcholine and the subsequent cholinergic crisis. While classic antidotes (atropine, pralidoxime, and benzodiazepine) have been proven to be effective in decreasing mortality rates, they do not target the delayed neurotoxicity associated with brain damage, neuronal inflammation, and apoptosis [11]. Another obstacle in the development of drugs expected to target the brain is that few can cross the blood–brain barrier (BBB). Thus, it is evident that an antidote capable of ameliorating neuroinflammation and neurodegeneration after AChE-I poisoning is needed.

The neuroprotective activity of 4R was discovered using ex vivo and in vivo models of neurodegeneration; for a review, see [5]. It was found that 4R suppressed the expression of behavioral sensitization to nicotine in rats [12] and decreased inflammation and apoptosis [3,13–15]. Rodents injected intra-cranially with 6-hydroxydopamine were shown to develop Parkinson’s disease (PD), and repeated injections of 4R nearly eliminated the behavioral, histological, and biochemical expression of this experimentally induced PD model [16]. Importantly, 4R was shown to protect ex vivo hippocampal slices against the N-methyl-D-aspartate (NMDA)-induced excitotoxicity via a nicotinic receptor-mediated mechanism that requires the inhibition of α7 nAChR and the indirect inhibition of α4β2 nAChRs, causing the activation of the PI3K/Akt anti-apoptotic signaling pathway [13,15]. Similarly, 4R ameliorated oxygen–glucose-deprivation-induced damage in rat hippocampal slices [17]. It was found that 4R protected neurons against irreversible inhibitors of AChE such as paraoxon [14,18] and DFP ex vivo and in vivo in Sprague Dawley rats [14,19]. These studies suggest that 4R could be an ideal candidate for use in protection against AChE-I poisoning. Nevertheless, the in vivo safety of this small molecule has not been tested.

Unlike the classical AChE-I antidotes, 4R not only crosses the BBB but also reaches a higher concentration in the brain than in plasma, suggesting that it is actively transported into the brain [20]. This study aimed to determine the safety of a single 4R s.c. dose given to male and female rats as a potential antidote against neurotoxic organophosphates.

## 2. Materials and Methods

### 2.1. Test Substance and Vehicle

The (1S,2E,4R,6R,7E,11E)-2,7,11-cembratriene-4,6-diol was obtained from El Sayed Research Foundation, University of Louisiana–Monroe, College of Pharmacy. The purity of 4R was >99% by the following analytical criteria: 1—1H NMR: Integration of H-6 Proton at δ 4.81 versus that of the 4S epimer (δ4.46); 2—13C NMR: C-6 in the 4R (δC 67.5) versus C-6 in the 4S (δC 66.0); 3—thin-layer chromatography (TLC): Rf value 0.42 (Si gel, n-hexane-Ethyl acetate 1:1).

Dimethyl sulfoxide (DMSO) and polyethylene glycol 400, NF (PEG 400) were obtained from VWR International, LLC (Brisbane, CA). In the following tests, 4R powder for s.c. administration was dissolved in a mixture of 10% DMSO and 90% PEG 400 to indicate dosing concentrations and formulate the final product. This formulation proved neuroprotective in vivo after exposure to irreversible AChE-inhibitor DFP [19].

Dose preparation was performed using an aseptic technique. The vehicle dose formulation was prepared by combining the appropriate amount of DMSO with PEG 400 and mixing it for 5 min using a magnetic stirrer until a clear, colorless solution was observed. The 4R dose formulations were prepared by firstly adding the appropriate amount of DMSO to the pre-weighed test article and mixing it for 2 min on a vortex mixer and sonicator until a clear, pale-yellow solution was observed. The appropriate amount of PEG 400 was then added and mixed for an additional 3 min until a clear, colorless to light yellow solution was observed.

### 2.2 Test Animals

Male and female Sprague Dawley (SD) rats from Charles River Laboratories, Hollister, CA, were delivered to the Animal Facility Center of the Stanford Research Institute (SRI) International (333 Ravenswood Avenue, Menlo Park, CA 94025, USA). The initial weight ranges were 221–277 g (males) and 123–202 g (females). The general procedures for animal care and housing were in accordance with the National Research Council (NRC) Guide for the Care and Use of Laboratory Animals (1996) and the Animal Welfare Standards incorporated in 9 CFR Part 3, 1991. The animals were quarantined for 3 days and housed 2–3 per cage. Hanging polycarbonate cages with hardwood chip bedding were utilized with a light cycle of 12 hr light/12 hr dark was applied at a temperature of 18 *°*C. Purina Certified Rodent Chow #5002 was provided ad libitum. For euthanasia, an overdose of sodium pentobarbital was administered via intraperitoneal injection. Every effort was made to minimize, if not eliminate, the pain and suffering in all of the animals used in this study.

### 2.3. Single-Dose Toxicity Study Design

The single-dose subcutaneous (s.c.) toxicity of 4R in rats was examined based on the fixed-dose procedure described in the “Single-dose acute toxicity testing for Pharmaceuticals, Industry Guidelines” from the FDA [21]. Forty rats (20 males and 20 females, age 8 weeks) were randomly divided into four groups/gender with five animals per group. The 4R dose was injected s.c. in the interscapular region via a slow bolus injection. This route of administration is proposed for clinical use in humans as it simplifies drug delivery and decreases the economic burden [22]. The 4R dose was previously shown to have neuroprotective properties at 6 mg/kg in various models of neurodegenerative diseases [16,17,19]. This study aimed to test the margin of safety of 4R in much higher doses.

Group 1 served as the control group and only received the vehicle (10% DMSO: 90% PEG 400). Groups 2 to 4 were treated by being injected with the test compound 4R at dose levels of 6, 24, and 98 mg/kg, respectively. The rats were observed for any symptoms of toxicity immediately after and during the following week. Observations for mortality, signs of illness, pain, injury, allergic responses, alterations in appearance, behavioral changes, such as ataxia and hyperactivity, general stimulation, and sedation were conducted every day. All of the observations were methodically recorded. After 7 days of monitoring, body weight was recorded, and the animals were euthanized via an overdose of sodium pentobarbital administered using intraperitoneal injection.

### 2.4. Gross Necropsy

At the end of the study, the animals fasted overnight before the euthanasia. External examinations of all of the body orifices and assessments of cranial, thoracic, and abdominal organs were performed. In addition, all gross pathology findings were recorded. The following tissues were collected and kept in 10% neutral buffered formalin: adrenal glands, aorta, bone (femur with a femorotibial joint), sternum, bone marrow, brain (fore-, mid-, and hindbrain), cecum, cervix, colon, duodenum, epididymis, esophagus, eyes, with optic nerve (fixed with formol alcohol preservative), heart, ileum, injection site (scapular region of the back), jejunum, kidneys, liver, lungs with bronchi, lymph nodes (mandibular and mesenteric) mammary glands (when present in a regular abdominal skin section, including the nipple and surrounding tissue), ovaries, pancreas, pituitary gland, prostate, rectum, salivary gland, mandibular, sciatic nerve, seminal vesicle (males), skeletal muscle, skin, and ventral abdomen, taken with mammary glands (females).

### 2.5. Histopathology

All tissues were fixed in 10% neutral buffered formalin containing neutral phosphate-buffered saline, trimmed, processed, embedded in paraffin, and sliced into standard 5 µm thick sections. Sections were stained with hematoxylin and eosin (H&E) according to routine histological techniques used by IDEXX laboratories. Each lesion was listed and coded by the most specific topographic and morphologic diagnoses, severity, and distribution, using the Systematized Nomenclature of Medicine (SNOMED) and the National Toxicology Program, Toxicology Data Management System (TDMS) as guides. A four-step grading system (minimal, mild, moderate, and marked) was used to define gradable lesions for comparison between the treated and control groups. Records of the gross findings for a specimen from postmortem observations were available to the pathologist when examining that specimen microscopically.

### 2.6. Blood Collection

Blood was collected on Days 3 and 8 from the retro-orbital sinus under 60% CO2/40% O2 anesthesia. Hematology samples were collected using EDTA tripotassium salt as an anticoagulant. No anticoagulant was used for serum chemistry samples. Animals did not fast before blood collection, and the data were obtained using the Cobas c-501 analyzer.

### 2.7. Statistical Analysis

Means and standard deviations were calculated for body weight (Labcat In-Life version 8.2) and clinical pathology (Labcat Clinical Pathology version 4.42). Body weight and clinical pathology data were evaluated using one-way analysis of variance ANOVA, followed by Dunnett’s test comparison test using Graph Pad Prism 5.0 (Graph Pad Prism, San Diego, CA, USA). For the clinical pathology data, values for parameters that were not within the detection threshold were included in the statistical evaluation. The level of significance was set at *p* < 0.05 to evaluate the vehicle control and treatment group.

## 3. Results

### 3.1. Body Weight and Clinical Signs of Toxicity

A 7-day post-observational period was applied after the 4R administration. There were no clinical signs related to toxicity. All animals survived until the day of their scheduled necropsy (Day 8). No apparent body weight changes associated with the administration of 4R were observed for any of the dose groups (Table 1).

Reactions at the injection site such as alopecia, discolored red fur, and eschar formation in the scapular area were found in the 4R-treated males and females. These findings were also observed with similar frequency and duration in the female control group, suggesting that these reactions may have been responses to irritation at the injection site and may be partially attributable to the application of 4R. Subcutaneous cavity formation characterized the site at which the injection bolus was placed in both the vehicle control and test-compound-treated rats. Clinical observations related to the injection site are summarized in Table 2.

### 3.2. Hematological Changes

As presented in Table 3, the hematological parameters, WBC, PLC, ANS, ALY, AMO, ABA, MCH, RDW, PLY, PMO, PEO, PBA, RET, MCV, HGB, and REA were not significantly different from the vehicle control group. However, on Day 3, the mean platelet volume (MPV) was increased (10%, *p* < 0.05) for the 6 mg/kg dose males and 24 mg/kg dose females compared with the controls (Table 3). On Day 8, the percent neutrophil count (PNS) increased (44%; *p* < 0.05) for the mid-dose males. However, this was attributed to the outlying result found in one of the rats in the male group, and it was not considered toxicologically relevant. The hematocrit (HCT) did not show relevant variations, and there was a significant decrease (*p* < 0.05) in mean absolute eosinophils (AEO) for the low- and high-dose females (53% and 58%, respectively). The mean reductions in AEO in females observed on Day 3 became insignificant by Day 8 (Table 3).

### 3.3. Biochemical Changes

The majority of the biochemical results concerning the male rats did not display significant changes caused by the 4R treatment (Table 4). However, on Day 3, the mean chloride level (CHL) was slightly increased (2%) for the mid-dose males and females compared with the controls. Nevertheless, this increase was not biologically significant. There was a decrease in triglyceride (TRI) levels for both males and females, particularly for the high-dose groups (51% and 30%, respectively). Yet, these reductions did not reach statistical significance. The sodium (SOD) level was increased by 1% (*p* < 0.05) and 2% (*p* < 0.01), respectively, for the mid- and high-dose males and increased by 2% (*p* < 0.01) for the high-dose females on Day 8. However, the sodium variations remained toxicologically irrelevant. The mean albumin (ALB) level was slightly decreased by 11% (*p*< 0.05) for the high-dose males, but this parameter was restored by Day 8. On Day 8, blood urea nitrogen (BUN) and creatinine (CRE) levels were significantly decreased in males 25% (*p* < 0.05) in the middle dose and 35% (*p* < 0.01) in the high dose, respectively (Table 4). The 4R dose did not affect the activity of the liver enzymes: aspartate aminotransferase (AST), alanine aminotransferase (ALT), and alkaline phosphatase (ALP) (Table 4). Thus, the absence of any alterations in the levels of hepatic enzymes supports the notion that 4R is not hepatotoxic.

### 3.4. Gross Organ and Histopathological Examination

Necropsy results show encrustation and thickening of the skin around the injection site in control animals and in male and female rats treated with 4R. Edema at the injection site was also observed for one low- and one high-dose female. Gross necropsy observations found discoloring, foci, and mottling in the lungs of the treated animals, which were also observed in the controls. These findings, which are typically related to euthanasia, were incidental, and were not considered to be related to the administration of 4R.

**Figure 1.**
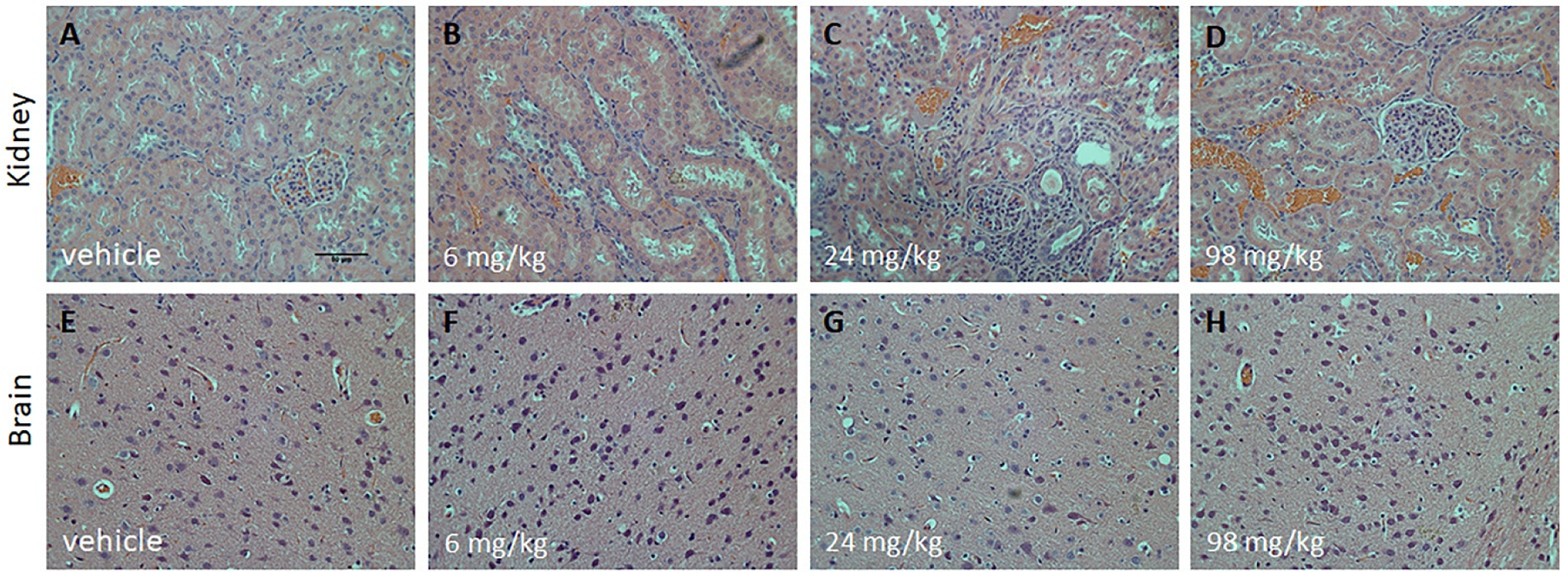
Histopathological analysis of H&E staining in male SD rats after a single dose of 4R. The blue/purple staining represents cells’ nuclei, the pale pink staining represents cells’ cytoplasm. (**A**–**D**) Normal microscopic structure of kidney and (**E**–**H**) brain of a male SD rat (25× magnification). Similar observations were obtained in female SD rats. Scale bar: 50 µm.

Histopathological observation of the stain tissues of the major organs (liver, spleen, heart, kidneys, and lungs) did not show any pathological lesions after the administration of 4R at all dose levels except at the skin injection site (discussed in Section 3.1). As shown in Figure 1, the brain and kidney sections were within normal limits in 4R- and vehicle-treated animals.

**Figure 2.**
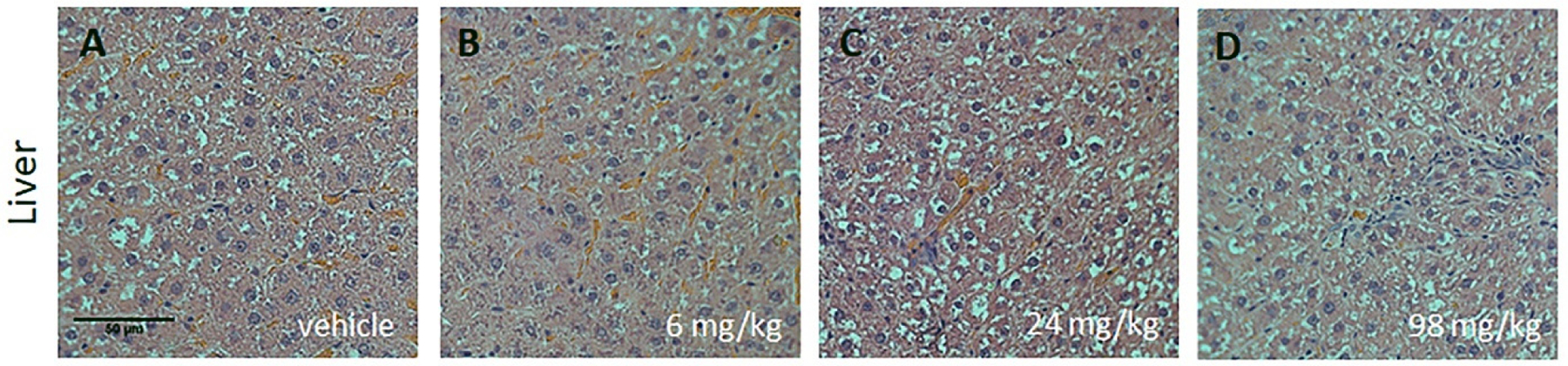
Histopathological analysis of H&E staining in the liver after a single dose of subcutaneous administration of 4R-cembranoid. Representative images from male SD rats (**A**–**D**). These sections show examples of hepatocellular hydropic change (pale cells) present in the vehicle- and 4R-treated groups (25× magnification). Similar observations were obtained in female SD rats. Scale bar: 50 µm.

Liver findings included the observation of hepatocellular hydropic changes in periportal areas (Figure 2). These morphological observations were all minimal to mild and were seen in the vehicle- and 4R-treated groups. Hepatocellular hydropic changes are common in young rats, and no finding was considered to be treatment-related. Hepatocellular hydropic changes (minimal to moderate) can reflect altered feeding status or altered metabolism, and it is reversible when the basis for its cause is discontinued [23].

## 4. Discussion

The threat of exposure to chemical neurotoxicants for civilians (agricultural and industry workers) and the military population has significantly increased [11,24]. The most likely warfare agents to be deployed are the irreversible organophosphorus inhibitors of AChE. Such exposure causes the accumulation of acetylcholine and the over-stimulation of muscarinic (central respiratory depression, bronchoconstriction, and hypotension) and nicotinic (peripheral muscle weakness, including respiratory) acetylcholine receptors. The classic pharmacotherapy against nerve agent poisoning is based on muscarinic antagonists (atropine) and reactivators of inhibited AChE (oximes) to reverse toxicity [25]. Nevertheless, survivors of nerve agents maintain persistent inflammatory statuses in the CNS, suggestive of an ongoing neurodegenerative process [26–29].

The present report tested the safety of 4R in male and female SD rats. The experimental design for a single s.c. dose of 4R was based on our previous investigation [19], where 4R complemented classical OP poisoning antidotes as it is able to cross the BBB [20] and protects against brain damage. The small molecule is a promising therapeutic candidate for use in the treatment of OP poisoning. This toxicity study placed the natural compound one step closer to clinical trial evaluation.

Tobacco-derived cembranoids are known to display cytotoxic, antifungal, antibacterial, anti-human immunodeficiency virus, anti-tumor, and neuroprotective properties [5]. However, to the best of our knowledge, this is the first report in which cembranoid safety was evaluated, and no severe clinically related adverse effects were detected. Several blood chemistry parameters and hematology findings were statistically different in subjects treated with 4R compared to vehicle controls. However, those differences were not significant and consistent enough to be clinically relevant. In general, the MPV increase observed in females treated with low and middle doses could be considered as a potential risk factor for stroke occurrence and a bad prognosis for stroke outcome [30,31]. However, the MPV increase was restored by Day 8, where the low-dose females had a mean 10% decrease in MPV, suggesting that this finding, although noteworthy, was not critical (Table 3).

In males treated with 4R, the blood chemistry results showed a significant decrease in CRE, BUN, and to a lesser extent, ALB levels (Table 4). Decreased BUN and CRE levels could be attributed to the reduced ingestion of food or severe hepatotoxicity [32]. Both factors were ruled out as there was no decrease in body weight. On the contrary, all of the treatment groups gradually gained weight in 7 days (Table 1), suggesting that the animals did not lose their appetites after drug exposure. Additionally, the levels of liver enzymes did not show signs of hepatotoxicity. On the contrary, elevation in BUN and CRE levels exceeded the normal range, which is typically associated with renal failure, but this was not detected during the post-observation period and the gross organ examination. Furthermore, changes in CRE and BUN values were not observed in females. Although these observations seem irrelevant from the perspective of toxicity, these findings should be considered in future studies.

In the histopathologic evaluation, no changes were attributed to the test substance. However, hepatocellular hydropic changes were detected (Figure 2). Minimal to mild hepatocellular hydropic changes are common in young rats and reflect an altered feeding status or altered metabolism. Most importantly, these changes are reversible [23].

We also observed reactions around the injection site, which are adverse occurrences commonly observed in the administration of various drugs and vehicles [33]. In males, the s.c. administration of 4R caused apparent adverse effects in the injection site, with prominent alopecia, discolored red fur, and eschar formation being observed (Table 2). However, these clinical observations were also present in the female controls, suggesting that skin irritation may have been caused by leakage of the vehicle formulation. We expect that the use of a different administration route of the compound, such as intraperitoneal (i.p.), intramuscular (i.m.), or intravenous (i.v.), would avoid the development of side effects on the skin.

PEG is the predominant vehicle in our formulation system as it has unusual solubility characteristics and a low degree of toxicity [34]. However, it is typical for subcutaneous dosages of PEG 300 and 400 administered to rats to produce blanching of the overlying dermis (within 24 h) and scab formation (48 h) after injection, along with increased vascularization and fibroblastic proliferation [35]. It is imperative to further assess suitable formulations and administration routes in future research.

## 5. Conclusions

The 4R-cembranoid did not appear to be toxic under the conditions provided in our study. All of the subjects, even those treated with the highest dose, survived the observation period without any overt sign of toxicity. The only significant adverse effects attributable to the compound were restricted to the s.c. injection site. This research provides evidence that supports the safety of 4R application in a rodent model. As acute toxicity testing is carried out to determine the effect of a single dose in a minimum of two mammalian species, including a nonrodent species, further testing on primates will define the use of 4R-cembranoid for clinical application in the treatment of neurotoxicity via OPs.

## Figures and Tables

**Table 1.** Body weight and mortality of Sprague Dawley rats after the single-dose 4R injection.

Body Weight (g)						
Sex	4R Dose (mg/kg)	Number Dosed	Day 1	Day 3	Day 8	Mortality (Dead/Total)
Male	0	5	253.8 ± 12.5	270.6 ± 15.1	312.6 ± 19.0	0% (0/5)
	6	5	257.0 ± 22.7	274.4 ± 20.3	305.8 ± 22.1	0% (0/5)
	24	5	258.6 ± 18.1	276.0 ± 19.7	312.5 ± 14.0	0% (0/5)
	98	5	252.2 ± 12.76	265.6 ± 10.6	299.6 ± 12.1	0% (0/5)
Female	0	5	171.4 ±13.4	185.2 ± 14.2	203.0 ± 14.0	0% (0/5)
	6	5	173.8 ± 13.0	181.4 ± 16.6	202.2 ± 20.5	0% (0/5)
	24	5	164.8 ± 32.1	185.8 ± 20.7	204.0 ± 17.6	0% (0/5)
	98	5	181.6 ± 7.7	191.6 ± 2.5	211.8 ± 4.8	0% (0/5)

VEH, vehicle administered 10% DMSO: 90% PEG. Data are expressed as means ± SD (n = 5/group). Statistics: one-way analysis of variance (ANOVA test) followed by Dunnett’s test, *p* < 0.05.

**Table 2.** Observations of the injection site regarding the fur and skin of male and female Sprague Dawley rats.

			Observation		
Sex	Dose (mg/kg)	Normal	Alopecia	Discolored—Red	Eschar Formation
Male	0	5	0	0	0
	6	2	3 (D5, D8)	3 (D8)	2 (D8)
	24	0	5 (D4, D8)	4 (D8)	5 (D6, D8)
	98	0	3 (D4, D8)	2 (D8)	5 (D6, D8)
Female	0	1	3 (D8)	2 (D8)	3 (D8)
	6	3	2 (D5, D8)	1 (D8)	2 (D6, D8)
	24	0	5 (D3, D8)	2 (D8)	5 (D6, D8)
	98	0	5 (D3, D8)	0	5 (D8)

Numbers represent the total quantity of examined animals with detected skin conditions in each category (normal, alopecia, discolored—red, and eschar formation). n = 5/group. D = day; the parentheses () contain the first and last day the observation was seen. Only animals that appeared normal on all days of observations are indicated as normal.

**Table 3.** Hematological changes after single-dose 4R administration in male and female Sprague Dawley rats.

Parameters	Day3				Day 8			
	Group (mg/kg)							
	0	6	24	98	0	6	24	98
**Male**								
WBC (×10^3^/µL)	16.55 ± 4.2	14.69 ± 1.9	15.74 ± 2.6	15.18 ± 1.7	15.15 ± 3.6	18.54 ± 5.9	16.02 ± 4.5	15.54 ± 1.5
PLC (×10^3^/µL)	1022 ± 160	905 ± 124.9	1105 ± 208.7	996 ± 111.3	1275 ± 176	1199 ± 296	1433 ± 360.8	1304 ± 154.9
AEO (×10^3^/µL)	0.28 ± 0.2	0.40 ± 0.1	0.16 ± 0.06	0.30 ± 0.05	0.32 ± 0.1	0.35 ± 0.1	0.35 ± 0.3	0.35 ± 0.1
ANS (×10^3^/µL)	3.67 ± 1.8	4.00 ± 1.8	5.12 ± 1.6	3.55 ± 0.9	2.72 ± 0.8	3.93 ± 2.3	5.18 ± 1.7	3.20 ± 1.1
ALY (×10^3^/µL)	11.85 ± 2.2	9.69 ± 1.2	9.67 ± 1.3	10.50 ± 1.5	11.57 ± 2.8	13.36 ± 3.8	9.89 ± 3.06	11.53 ± 2.1
AMO (×10^3^/µL)	0.67 ± 0.2	0.53 ± 0.2	0.73 ± 0.2	0.77 ± 0.1	0.46 ± 0.2	0.70 ± 0.2	0.53 ± 0.2	0.41 ± 0.1
ABA (×10^3^/µL)	0.08 ± 0.05	0.07 ± 0.01	0.06 ± 0.02	0.06 ± 0.01	0.07 ± 0.02	0.10 ± 0.06	0.06 ± 0.02	0.06 ± 0.01
MCH (pg)	19.3 ± 0.3	19.8 ± 0.7	19.8 ± 0.2	19.5 ± 0.4	19.7 ± 0.2	19.9 ± 0.7	20.2 ± 0.3	19.7 ± 0.3
PNS (%)	21.2 ± 5.8	26.4 ± 10.4	32.0 ± 7.7	23.3 ± 5.03	17.9 ± 2.4	20.1 ± 7.8	32.1 ± 7.7 *	20.7 ± 7.4
HCT (%)	42.2 ± 2.2	41.1 ± 1.1	40.7 ± 0.9	41.4 ± 1,8	41.0 ± 3.9	44.7 ± 3.1	42.4 ± 3.1	43.8 ± 3.8
RDW (%)	13.5 ± 0.3	13.2 ± 0.9	13.0 ± 0.6	12.8 ± 0.4	16.5 ± 1.08	14.8 ± 1.8	15.2 ± 0.9	15.2 ± 1.2
PLY (%)	72.7 ± 6.7	66.9 ± 12.1	62.0 ± 8.08	69.2 ± 5.7	76.4 ± 3.1	73.8 ± 8.1	62 ± 8.3	73.9 ± 8.9
PMO (%)	4.0 ± 0.6	3.5 ± 1.5	4.6 ± 0.9	5.1 ± 0.7	3.0 ± 0.8	3.6 ± 0.5	3.5 ± 1.5	2.7 ± 0.9
PEO (%)	1.7 ± 1.1	2.7 ± 0.7	1.0 ± 0.4	2.0 ± 0.4	2.3 ± 1.2	2.0 ± 0.8	2.0 ± 1.3	2.2 ± 1.03
PBA (%)	0.5 ± 0.2	0.5 ± 0.09	0.4 ± 0.12	0.4 ± 0.1	0.5 ± 0.09	0.5 ± 0.1	0.4 ± 0.05	0.4 ± 0.07
RET (%)	5.87 ± 0.6	6.26 ± 1.4	5.90 ± 0.8	5.27 ± 0.2	12.20 ± 0.8	9.99 ± 1.6	10.71 ± 2.2	10.98 ± 1.5
MCV (fL)	64.9 ± 0.6	65.2 ± 2.3	66.1 ± 1.0	65.3 ± 1.6	66.8 ± 0.9	67.6 ± 1.8	67.2 ± 0.8	66.6 ± 1.42
MPV (fL)	8.3 ± 0.5	9.2 ± 0.6 *	8.5 ± 0.4	9.0 ± 0.2	8.2 ± 0.4	8.6 ± 0.6	8.2 ± 1.3	8.5 ± 0.3
HGB (g/dL)	12.5 ± 0.7	12.4 ± 0.5	12.2 ± 0.2	12.4 ± 0.5	12.1 ± 1.1	13.2 ± 1.08	12.8 ± 0.2	13.0 ± 1.2
REA (10^9^/L)	382.6 ± 56	396 ± 86.3	362.6 ± 50.04	336.2 ± 27	745.6 ± 58	656.4 ± 79	675.3 ± 124	720.3 ± 93.1
RBC (10^6^/µL)	6.50 ± 0.2	6.30 ± 1.1	6.15 ± 0.081	6.34 ± 0.27	6.13 ± 0.5	6.62 ± 0.5	6.32 ± 0.1	6.58 ± 0.5
**Female**								
WBC (×10^3^/µL)	16.38 ± 2.1	15.75 ± 2.9	17.27 ± 3.08	15.00 ± 2.0	12.83 ± 6.3	14.88 ± 3.3	14.65 ± 2.3	13.28 ± 1.7
PLC (×10^3^/µL)	1012 ± 156	1207 ± 114.8	1010 ± 137.4	1125 ± 74.3	1394 ± 349	1479 ± 235	1273 ± 231	1626 ± 129
AEO (×10^3^/µL)	0.36 ± 0.1	0.17 ± 0.058 *	0.24 ± 0.047	0.15 ± 0.103 *	0.27 ± 0.1	0.22 ± 0.06	0.25 ± 0.084	0.18 ± 0.1
ANS (×10^3^/µL)	3.72 ± 1.0	2.88 ± 1.2	3.07 ± 0.8	2.60 ± 0.7	2.11 ± 1.1	2.85 ± 1.1	2.21 ± 0.5	2.39 ± 0.9
ALY (×10^3^/µL)	11.50 ± 1.7	12.03 ± 2.8	13.26 ± 2.5	11.22 ± 2.1	10.06 ± 4.9	11.38 ± 2.9	11.70 ± 2.1	10.32 ± 1.2
AMO (×10^3^/µL)	0.71 ± 0.2	0.60 ± 0.2	0.60 ± 0.1	0.96 ± 0.3	0.33 ± 0.3	0.37 ± 0.2	0.43 ± 0.1	0.34 ± 0.06
ABA (×10^3^/µL)	0.09 ± 0.02	0.07 ± 0.03	0.10 ± 0.04	0.05 ± 0.03	0.06 ± 0.04	0.06 ± 0.02	0.08 ± 0.01	0.04 ± 0.01
MCH (pg)	20.1 ± 0.5	19.6 ± 0.6	19.9 ± 1.05	20.0 ± 0.8	20.6 ± 0.3	20.0 ± 0.5	20.7 ± 1.1	20.5 ± 0.4
PNS (%)	22.7 ± 5.5	18.5 ± 8.8	17.8 ± 4.2	17.5 ± 5.8	16.8 ± 5.4	19.3 ± 7.4	15.2 ± 4.4	17.8 ± 4.9
HCT (%)	43.0 ± 2.1	40.7 ± 1.0	39.8 ± 2.2 *	42.2 ± 1.1	40.2 ± 4.7	39.5 ± 1.5	40.5 ± 2.2	40.7 ± 1.3
RDW (%)	11.9 ± 0.4	12.5 ± 1.0	11.8 ± 0.4	11.9 ± 0.4	15.3 ± 0.7	15.4 ± 1.4	16.3 ± 2.0	16.2 ± 1.9
PLY (%)	70.2 ± 6.0	76.1 ± 8.9	76.7 ± 4.4	74.4 ± 5.0	78.3 ± 4.7	76.4 ± 7.5	79.7 ± 5.7	77.9 ± 5.2
PMO (%)	4.4 ± 1.3	3.8 ± 0.6	3.5 ± 1.0	6.5 ± 2.8	2.2 ± 0.9	2.3 ± 0.8	2.9 ± 0.9	2.6 ± 0.6
PEO (%)	2.2 ± 0.8	1.2 ± 0.5	1.5 ± 0.4	1.0 ± 0.7	2.3 ± 1.3	1.6 ± 0.7	1.8 ± 0.6	1.3 ± 0.6
PBA (%)	0.5 ± 0.1	0.4 ± 0.1	0.5 ± 0.1	0.3 ± 0.1	0.4 ± 0.1	0.4 ± 0.09	0.5 ± 0.1	0.4 ± 0.05
RET (%)	4.12 ± 0.7	5.43 ± 0.8	3.89 ± 1.7	4.82 ± 1.3	12.99 ± 1.8	11.85 ± 1.5	13.39 ± 4.0	12.93 ± 2.7
MCV (fL)	65.3 ± 1.3	64.6 ± 1.3	65.0 ± 3.3	65.8 ± 2.4	68.4 ± 1.1	67.1 ± 1.4	68.9 ± 3.1	68.7 ± 1.8
MPV (fL)	7.8 ± 0.4	8.0 ± 0.7	8.7 ± 0.3 *	8.5 ± 0.2	7.9 ± 0.6	7.1 ± 0.3 *	8.2 ± 0.4	7.8 ± 0.3
HGB (g/dL)	13.2 ± 0.6	12..3 ± 0.3	12.2 ± 0.8	12.8 ± 0.4	12.1 ± 1.4	11.8 ± 0.3	12.2 ± 0.7	12.2 ± 0.3
REA (10^9^/L)	271.9 ± 56	316.6 ± 57.6	235.5 ± 100.2	307.7 ± 82	755.7 ± 87	698.3 ± 93	791.0 ± 244	765.5 ± 160

WBC: white blood cell count; PLC: platelet count; AEO: absolute eosinophils; ANS: total neutrophil; ALY: total lymphocyte; AMO: total monocyte; ABA: total basophil; MCH: mean corpuscular hemoglobin; PNS: percent neutrophil count; HCT: hematocrit; RDW: red blood cell distribution width; PLY: percent lymphocyte; PMO: percent monocyte; PEO: percent eosinophil; PBA: percent basophil; MCV: mean corpuscular volume; MPV: mean platelet volume; HGB: hemoglobin; RET: reticulocyte count percent. Data are expressed as means ± SD (n = 5/group).

* Significantly different from the vehicle control group (*p* < 0.05).

**Table 4.** Clinical biochemistry after a single s.c. 4R dose in male and female Sprague Dawley rats.

Parameters	Day 3				Day 8			
	Group (mg/kg)							
	0	6	24	98	0	6	24	98
**Male**								
ALB (×10^3^/µL)	3.8 ±0.82	3.8 ± 0.1	3.6 ± 0.16	3.4 ± 0.16 *	4.0 ± 0.31	4.0 ± 0.46	3.9 ± 0.13	3.9 ± 0.27
BUN (×10^3^/µL)	14 ± 2.0	16 ± 1.9	14 ± 1.9	15 ± 1.9	20 ± 2.9	19 ± 2.8	15 ± 2.4 *	13 ± 0.8 **
CRE (×10^3^/µL)	0.24 ± 0.01	0.22 ± 0.02	0.24 ± 0.02	0.22 ± 0.03	0.29 ± 0.01	0.27 ± 0.01	0.22 ± 0.02 **	0.23 ± 0.01 **
AST (U/L)	90 ± 9.0	86 ± 4.2	99 ± 10.1	90 ± 8.6	77 ± 0.4	77 ± 5.1	83 ± 10.5	81 ± 9.3
ALT (U/L)	48 ± 5.2	51 ± 7.0	54 ± 9.0	45 ± 4.0	43 ± 2.2	44 ± 6.0	43 ± 6.5	42 ± 5.0
ALP (U/L)	255 ± 47	227 ± 42.3	224 ± 27.0	234 ± 16.7	257 ± 35.4	215 ± 33.8	228 ± 44.5	232 ± 19.1
SOD (mEq/L)	145 ± 1.3	146 ± 0.4	147 ± 0.8	146 ± 1.1	142 ± 1.0	142 ± 0.5	144 ± 1.0 **	145 ± 1.1 **
POT (mEq/L)	6.6 ± 0.39	6.4 ± 0.28	6.4 ± 0.17	6.4 ± 0.28	6.8 ± 0.40	6.8 ± 0.40	6.2 ± 0.48	6.6 ± 0.46
CHL (mEq/L)	100 ± 1.1	100 ± 1.4	102 ± 1.6 *	102 ± 0.9	100 ± 1.5	99 ± 1.3	100 ± 1.7	100 ± 1.4
CHO (mg/dL)	92 ± 10.3	79 ± 19.0	80 ± 16.3	86 ± 12.0	75 ± 8.4	70 ± 12.8	75 ± 15.7	92 ± 10.3
TRI (mg/dL)	153 ± 52.3	128 ± 55.7	103 ± 38.3	75 ± 17.4	145 ± 22.9	120 ± 26.1	122 ± 84.3	126 ± 24.5
GLU (mg/dL)	132 ± 7.9	133 ± 14.4	137 ± 10.3	136 ± 6.5	175 ± 49.9	196 ± 35.1	143 ± 16.6	132 ± 7.9
CAL (mg/dL)	12.0 ± 0.34	12.0 ± 0.23	12.1 ± 0.29	12.6 ± 0.4 *	12.4 ± 0.75	13.0 ± 0.09	12.8 ± 0.19	12.0 ± 0.34
PHO (mg/dL)	11.2 ± 0.26	11.3 ± 0.97	11.1 ± 0.56	11.9 ± 0.38	12.8 ± 1.73	13.2 ± 1.35	12.3 ± 0.92	11.2 ± 0.26
TPR (mg/dL)	5.7 ± 0.28	5.6 ± 0.18	5.5 ± 0.24	5.5 ± 0.34	5.8 ± 0.32	5.9 ± 0.51	5.8 ± 0.24	5.7 ± 0.28
GLO (g/dL)	1.9 ± 0.21	1.9 ± 0.15	1.9 ± 0.23	2.2 ± 0.32	1.8 ± 0.09	1.9 ± 0.11	1.9 ± 0.29	1.9 ± 0.21
**Female**								
ALB (×10^3^/µL)	4.2 ± 0.27	4.2 ± 0.24	3.9 ± 0.35	3.9 ± 0.15	4.4 ± 0.4	4.6 ± 0.22	4.4 ± 0.19	4.2 ± 0.19
BUN (×10^3^/µL)	14 ± 2.0	15 ± 1.9	14 ± 2.5	15 ± 3.5	17 ± 2.3	16 ± 2.3	16 ± 3.9	14 ± 1.4
CRE (×10^3^/µL)	0.22 ± 0.03	0.23 ± 0.03	0.23 ± 0.03	0.22 ± 0.03	0.29 ± 0.09	0.25 ± 0.06	0.28 ± 0.03	0.24 ± 0.02
AST (U/L)	90 ± 9.0	86 ± 4.2	99 ± 10.1	90 ± 8.6	77 ± 0.4	77 ± 5.1	83 ± 10.5	81 ± 9.3
ALT (U/L)	48 ± 5.2	51 ± 7.0	54 ± 9.0	45 ± 4.0	43 ± 2.2	44 ± 6.0	43 ± 6.5	42 ± 5.0
ALP (U/L)	255 ± 47.0	227 ± 42.3	224 ± 27.0	234 ± 16.7	257 ± 35.4	215 ± 33.8	228 ± 44.5	232 ± 19.1
SOD (mEq/L)	145 ± 1.3	144 ± 1.2	146 ± 0.7	146 ± 1.1	142 ± 0.7	142 ± 1.7	143 ± 1.1	145 ± 0.9 **
POT (mEq/L)	6.2 ± 0.40	6.4 ± 0.27	6.3 ± 0.67	6.5 ± 0.41	6.5 ± 030	6.5 ± 0.46	6.3 ± 0.67	6.6 ± 0.50
CHL (mEq/L)	100 ± 0.8	100 ± 1.5	102 ± 0.8 *	101 ± 0.9	100 ± 2.1	99 ± 0.8	102 ± 1.2	100 ± 0.5
TRI (mg/dL)	91 ± 20.4	74 ± 29.9	73 ± 13.3	64 ± 12.2	90 ± 22.9	84 ± 38.7	66 ± 14.1	78 ± 42.4
CHO (mg/dL)	85 ± 15.9	82 ± 6.3	72 ± 6.5	75 ± 9.5	79 ± 14.9	80 ± 4.5	78 ± 8.5	76 ± 12.2
GLU (mg/dL)	130 ± 13.4	133 ± 6.3	139 ± 8.2	130 ± 4.4	164 ± 58.3	149 ± 42.7	159 ± 27.4	125 ± 10.2
CAL (mg/dL)	12.1 ± 0.29	12.2 ± 0.41	11.9 ± 0.25	12.6 ± 0.42	12.5 ± 0.56	12.7 ± 0.67	12.7 ± 0.30	12.8 ± 0.30
PHO (mg/dL)	10.5 ± 0.5	10.0 ± 1.05	10.0 ± 0.27	11.0 ± 0.67	11.7 ± 2.54	11.5 ± 1.62	12.3 ± 1.80	11.2 ± 0.55
GLO (g/dL)	1.9 ± 0.17	1.7 ± 0.11	1.8 ± 0.26	2.0 ± 0.23	1.6 ± 0.15	1.5 ± 0.17	1.7 ± 0.20	1.8 ± 0.19

Abbreviations: ALB: albumin; BUN: blood urea nitrogen, CRE: creatinine; SOD: sodium; POT: potassium; CHL: chloride; CHO: cholesterol, TRI: triglycerides; GLU: glucose; AST: aspartate aminotransferase; ALT: alanine aminotransferase; ALP: alkaline phosphatase; CAL: calcium; PHO: phosphorus; TRP: protein, total; GLO: globulin. Data are expressed as means ± SD (n = 5/group).

* Significantly different from the vehicle control group was labeled at * *p* < 0.05 and

** *p* < 0.01 comparison with the vehicle control group.
